# H&E image analysis pipeline for quantifying morphological features

**DOI:** 10.1016/j.jpi.2023.100339

**Published:** 2023-10-05

**Authors:** Valeria Ariotta, Oskari Lehtonen, Shams Salloum, Giulia Micoli, Kari Lavikka, Ville Rantanen, Johanna Hynninen, Anni Virtanen, Sampsa Hautaniemi

**Affiliations:** a Research Program in Systems Oncology, Research Programs Unit, Faculty of Medicine, University of Helsinki, 00014 Helsinki, Finland; b Department of Obstetrics and Gynaecology, University of Turku and Turku University Hospital, 200521 Turku, Finland; c Department of Pathology, University of Helsinki and HUS Diagnostic Center, Helsinki University Hospital, 00029 Helsinki, Finland

**Keywords:** Digital pathology, Whole-slide images, Instance segmentation, Feature extraction, Ovarian high-grade serous carcinoma, Ploidy

## Abstract

Detecting cell types from histopathological images is essential for various digital pathology applications. However, large number of cells in whole-slide images (WSIs) necessitates automated analysis pipelines for efficient cell type detection. Herein, we present hematoxylin and eosin (H&E) Image Processing pipeline (HEIP) for automatied analysis of scanned H&E-stained slides. HEIP is a flexible and modular open-source software that performs preprocessing, instance segmentation, and nuclei feature extraction. To evaluate the performance of HEIP, we applied it to extract cell types from ovarian high-grade serous carcinoma (HGSC) patient WSIs. HEIP showed high precision in instance segmentation, particularly for neoplastic and epithelial cells. We also show that there is a significant correlation between genomic ploidy values and morphological features, such as major axis of the nucleus.

## Introduction

Histopathological examination of formalin-fixed, paraffin-embedded (FFPE) tissue samples is the cornerstone of cancer diagnosis. The most common staining of the tissue samples is hematoxylin and eosin (H&E), which has been used for more than a century for deducing tumor morphology, cell types, invasion, mitotic activity, and tumor grade.^1^^,^^2^ With the development of high-resolution scanners, it has become possible to digitize histopathological samples, which enables the use of machine learning methods on H&E slides. These methods can assist pathologists in diagnostic tasks^3^ and extract multi-parametric features from the histological phenotype that may not be readily accessible to the human eye.^4^

In recent years, deep learning (DL) methods have been used for various predicting tasks on H&E images without the need to segment and annotate cell types.^5^^,^^6^ However, for some computational pathology applications and approaches, such as combining cell morphology to genomics data, it is necessary to extract and annotate cell types from digitalized slides.^7^, ^8^, ^9^, ^10^ These approaches enable computational analysis of the morphological features for tens of thousands of cells within a single H&E slide, as well as the spatial distribution of cells.^11^, ^12^, ^13^, ^14^ Cell segmentation and annotation tasks are challenging because of the diversity of nuclei characteristics, the presence of overlapping cells, variance in tissue staining, and background noise.

Various methods for cell nuclei classification have been proposed, such as support vector machine^10^ and AdaBoost classifiers.^15^ Other DL-based approaches have been utilized for nuclei detection, such as the spatially constrained convolutional neural network (CNN)^16^ and the multi-task CNN for simultaneous nuclei segmentation and classification.^17^^,^^18^ While these approaches perform well on different microscopic image modalities, they lack the necessary flexibility to be trained with a variety of training routines. Additionally, their model architectures lack the flexibility to be adjusted or expanded for inference latency, *i.e.,* the duration between input and output of a model, or segmentation performance gains, making impossible to optimize the latency-performance trade-off of the models. This type of modifiability is necessary in digital pathology, where hundreds of gigapixel-sized whole-slide images (WSIs) are processed.

To address the need of detecting cell types from digitalized H&E slides and extract their morphological features, we developed an open-source computational framework, called H&E Image Processing pipeline (HEIP). HEIP has modular design, which makes it easy to be modified and adjusted to reduce inference latency. The core of HEIP is a modified version of the HoverNet architecture^17^ with a post-processing approach that enables the simultaneous segmentation and annotation of cells from digitalized H&E WSIs (subsequently H&E images).

To demonstrate the utility of HEIP, we analyzed H&E images from ovarian high-grade serous carcinoma (HGSC) patients. HGSC is the most common and aggressive subtype of epithelial ovarian cancer that is typically diagnosed at an advanced stage with widespread metastasis in the peritoneal cavity. Even though most patients have an excellent initial response, the 5-year survival rate in HGSC is less than 40%.^19^

Herein, we evaluate HEIP’s instance segmentation performance with two HGSC datasets, focusing on cell classification. We also evaluate HEIP's performance in different sites: tubo-ovarian tumors (uterine adnex, ovary, and tubes), and intra-abdominal metastases (omentum and peritoneum). To demonstrate the utility of HEIP, we conducted an exemplifying analysis to explore the association of the morphological nuclear features and the ploidy values, which in a cell correspond to a complete set of chromosomes, computed from whole-genome sequencing data of patients with HGSC.

## Material and methods

### Patient cohorts

The H&E images used in this study originated from the DECIDER observational clinical trial and PanNuke study.

Firstly, the DECIDER dataset contains image data from HGSC patients participating in the longitudinal, multiregional observational study DECIDER (Multi-layer Data to Improve Diagnosis, Predict Therapy Resistance and Suggest Targeted Therapies in HGSOC; ClinicalTrials.gov identifier: NCT04846933). The image data used herein consists of scanned images of H&E stained slides from archival formalin-fixed paraffin-embedded (FFPE) tissue blocks collected at the time of diagnosis both for routine diagnostic and research purposes. The archival diagnostic slides were obtained from Auria biobank. The preparation of the research-purpose FFPE block was carried out by the Histology core facility at the Institute of Biomedicine, University of Turku, Finland. All slides were stained at the department of pathology in Turku University Hospital. The scanning of the images was done by Auria Biobank (University of Turku) and the slides were stored in OMERO database.^20^ The DECIDER data were divided into training and validation datasets (see below).

Secondly, we used the PanNuke dataset^21^ in the training stage. The PanNuke dataset is a publicly available dataset of automatically generated nuclei instance segmentation and classification, from 19 different tissue types and cancer, from more than 20K patches at different magnifications.^21^

Training dataset: For the instance segmentation method, we trained the model using a dataset of 13 H&E images from 13 HGSC patients from the DECIDER cohort. A total of 197 regions of interest (ROIs) were selected from 13 H&E images by a pathologist (A.V.). The ROIs were chosen from various tissue types and had varying dimensions, with a focus on selecting regions that contained different cell types. Subsequently, the cells in the ROIs were annotated by A.V with the train-in-the-loop approach,^22^ using the software QuPath^23^ resulting in 36 093 cell annotations. The cell types included were neoplastic, inflammatory, connective, non-neoplastic epithelial, and dead cells. Additionally, we included 205 343 cell annotations from the PanNuke dataset^21^ in the training dataset.

Validation datasets: The model was validated with 2 subsets of images from the DECIDER cohort. The validation set images were not used in the training stage and were annotated with the train-in-the-loop approach^22^ by a pathologist (A.V.). The first validation dataset, "CellTypeValidation", was designed to assess instance segmentation performance across the cell types. The second validation dataset, "TumorSiteCellValidation", was designed to assess HEIP performance in different tumor sites.

The CellTypeValidation dataset consisted of 20 human selected ROIs extracted from H&E images of 19 HGSC samples, totaling 9461 train-in-the-loop^22^ annotated cell instances. The distribution of cell types across the analyzed regions is as follows: 38% of neoplastic cells, 18% of inflammatory cells, 36% of connective cells, 8% of epithelial cells, and 0.1% of dead cells. The majority of neoplastic cells were in ROIs located in the peritoneum, omentum, uterus, mesenterium, and subcutaneous tissue. In contrast, connective cells were more abundant in ROIs from tubo-ovarian regions, while epithelial cells were more prevalent in ROIs from bowel tissue.

The TumorSiteCellValidation dataset was comprised of 36 ROIs located at the tumor–stroma interface of 18 randomly selected H&E images, including omental (6), peritoneal (6), and tubo-ovarian (6) tumors, from an equal number of HGSC patients. We selected 2 1000 × 1000 pixel ROIs from each H&E image. The distribution of cell types is primarily composed of neoplastic cells (58%), followed by connective cells (26%) and inflammatory cells (16%). Neoplastic cells accounted for over 50% of each tissue type, while connective cells accounted for over 20%, reaching 33% in the case of peritoneum. Dead and epithelial cells were excluded from the analysis as their number in the ROIs was non-existent or too small for reliable analysis

We also show an example of a possible downstream analysis by calculating correlation between features extracted from images and genomic ploidy values. The ploidy association dataset contains an independent subset of patients in the DECIDER cohort. The samples in ploidy vs. feature correlation analysis were matched, *i.e.*, the H&E image and whole-genome sequencing sample are taken from the adjacent locations of the same tumor piece. We obtained 47 digitalized H&E slides from 23 HGSC patients with this criterion. The H&E images were obtained from omental (18), peritoneal (12), and tubo-ovarian (17) tumors.

### Image preprocessing

The H&E images were scanned in MIRAX format with 20× magnification. To prepare the images for analysis, we used a Python library called HistoPrep.^24^ HistoPrep was employed to identify and segment tissue areas from H&E images into patches. Additionally, patches with insufficient information or a low signal-to-noise ratio were excluded using a series of filters. The H&E images were partitioned into patches with dimensions of 1250 × 1250 pixels, and for each image, the patches were saved in a separate folder in PNG format. The number of patches varied depending on the size of the tissue and the filtering applied, ranging from hundreds to thousands per image.

### Deep learning instance segmentation model

A deep learning approach was developed to segment and classify the nuclei. The model is a multi-task CNN, loosely based on the HoVer-net architecture.^17^ Similar to HoVer-Net, the architecture comprises a shared encoder and 3 distinct task-specific decoders with distinct output tasks. However, instead of using the post-processing method used by the HoVer-Net model, we opted for the Omnipose post-processing approach^25^ due to its better overall segmentation performance, as demonstrated in Table S1.

The segmentation and classification performances were evaluated using the following metrics: segmentation quality (SQ), detection quality (DQ), and panoptic quality (PQ). SQ is calculated as the normalized mean of the Intersection over Union (IoU), which measures the quality of the object delineation. DQ, also known as F1-score, is the harmonic mean of precision and recall and measures how well countable objects are detected from the background. PQ is defined as the product of DQ and SQ and quantifies the performance of instance segmentation in a unified manner. The formulas for these metrics are as follows:^26^

$$\mathrm{DQ}=\frac{\left|\mathrm{TP}\right|}{\left|\mathrm{TP}\right|+\frac{1}{2}\left|\mathrm{FP}\right|+\frac{1}{2}\left|\mathrm{FN}\right|}$$

$$\mathrm{SQ}=\frac{\sum_{\left(p,g\right)\in \mathrm{TP}}\mathrm{IoU}\left(p,g\right)}{\left|\mathrm{TP}\right|}$$

$$\mathrm{PQ}=\mathrm{DQ}\times \mathrm{SQ}=\frac{\sum_{\left(p,g\right)\in \mathrm{TP}}\mathrm{IoU}\left(p,g\right)}{\left|\mathrm{TP}\right|+\frac{1}{2}\left|\mathrm{FP}\right|+\frac{1}{2}\left|\mathrm{FN}\right|}$$

where *TP*, *FP*, and *FN* denote the true-positive, false-positive and false-negative, respectively. *IoU* denotes the intersection-over-union and was set to 0.5.

In order to provide a more precise assessment of the HEIP instance segmentation, we employed estimated confidence intervals (CIs) using a bootstrapping approach.^27^ Bootstrapping employs resampling the validation dataset multiple times (*n* = 200). We derived confidence bounds by extracting percentiles from the resulting bootstrap distribution.^28^

After instance segmentation, a json file, in geojson format, is generated containing the coordinates of each detected nucleus as polygons. HEIP then extracts various nuclei features from the json file, including area, volume, solidity, eccentricity, minor axis, major axis, aspect ratio, and perimeter. Additionally, it estimates the percentage of cell types and Shannon index entropy values.^29^ The definition of each feature is detailed in Table S2.

### Whole genome sequencing

We conducted a WGS analysis to investigate the correlation between nuclear cell characteristics, as extracted using HEIP, and ploidy. The approach used for this analysis is consistent with the methodology outlined in the Methods section of Lahtinen et al.^30^

### Copy number calling, ploidy, and purity estimation

Copy number calling was conducted on 23 patients using the Hartwig Medical Foundation toolkit, with genomic breakpoints and breakends extracted using the Genomic Rearrangement Identification Software Suite (GRIDSS).^31^

B-allele frequency (BAF) was calculated with AMBER (https://github.com/hartwigmedical/hmftools/tree/master/amber/) using heterozygous single nucleotide polymorphismSNPGATK Mutect2;^32^ and read depth extracted using COBALT (https://github.com/hartwigmedical/hmftools/tree/master/cobalt/). PURity and PLoidy Estimator (Purple)^33^ was used to estimate the copy-number profile, purity, and ploidy by combining BAF, read depth, filtered breakpoints, and somatic mutations.

The model used to calculate purity and ploidy selected the most parsimonious solution among a grid of possible combinations using a fit score. The fit score was determined by a deviation penalty, event penalty multiplier, and somatic deviation penalty. The deviation penalty penalized solutions requiring subclonality to explain copy number patterns, while the event penalty aimed to disfavor the number of alterations required to pass from normal diploid chromosomes to observed minor and major allele copy numbers. Additionally, combinations of [purity; ploidy] values that violated the rule of somatic variants were penalized.

### Statistical analyses

HEIP extracts features for each individual nucleus present in the tissue samples, resulting in data from hundreds or thousands of nuclei features. To summarize the data and provide representative statistical measurements for each sample, we employed the median and variance. Subsequently, the correlation between the median and the variance of each morphological feature (area, volume, major axis, and perimeter) of neoplastic nuclei and the corresponding ploidy value of the samples was computed. The Spearman correlation was used to calculate correlation. Analysis of variance (ANOVA) was used to investigate the correlation between ploidy and the 3 tumor locations: omentum, tubo-ovarian, and peritoneum. All statistical analyses were performed using R software (version 4.2.1).

## Results

### Overview of the HEIP pipeline

**Fig. 1:**
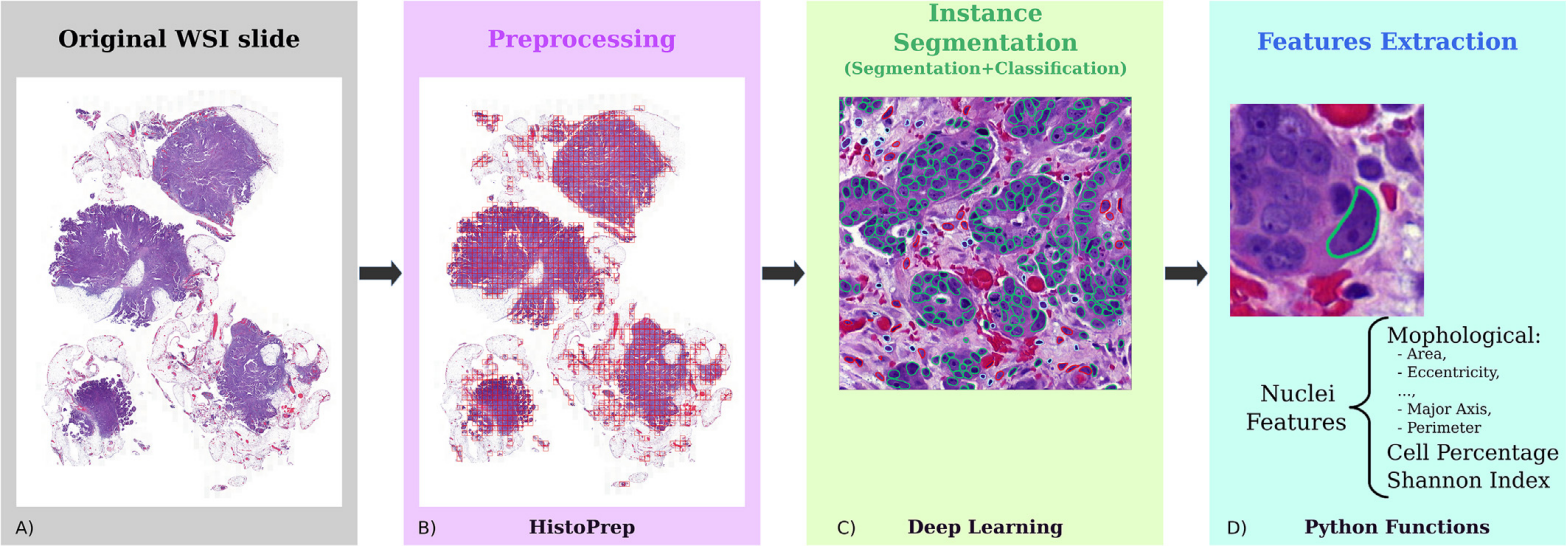
HEIP schematic workflow. HEIP is a comprehensive software for processing H&E images in order to detect cell nuclei and their morphological features. Panel A: The input to HEIP is a digitized H&E image. Panel B: Preprocessing step is done with HistoPrep. Patches are visible in red. Panel C: Nuclei are detected with deep learning instance segmentation. Panel D: Cell nuclei feature extraction, such as morphological features, cell percentages, and Shannon Index.

The HEIP pipeline is designed to extract cell nuclei and their morphological nuclear features from H&E images using a DL-based segmentation model as illustrated in Fig. 1. Briefly, the pipeline is based on two customizable steps. The first step processes the H&E images to be amenable for analyses. The second step consists of instance segmentation, which is further divided into cell segmentation and classification steps. Additionally, various Python functions, such as shapely geometry functions, were utilized to extract morphological nuclear features from cell nuclei as well as cell percentages and Shannon Index, which measures entropy. The HEIP pipeline is designed and implemented to be modular and is therefore easy to modify.

### Instance segmentation results

**Fig. 2:**
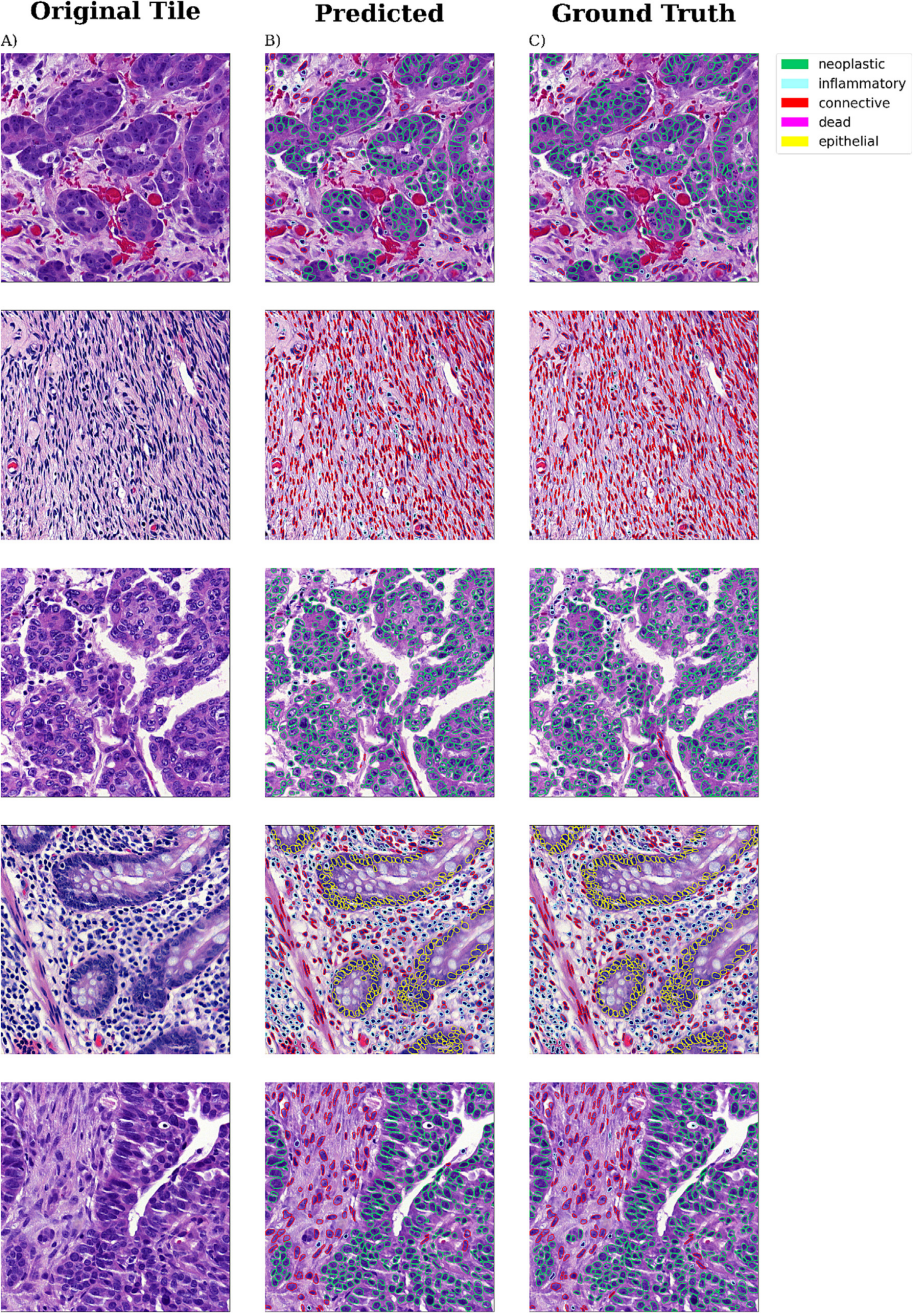
Instance segmentation examples. Five examples of performance of HEIP, the ROIs were chosen from various tissue types (tubo-ovarian, omentum, bowel, and peritoneum), focusing on different cell types. Panel A: Original tiles from an H&E image. Panel B: Cell classification results by HEIP. Panel C: Ground truth of the nuclei, borders, and types by pathologist.

Upon visual inspection, the instance segmentation results were very close to the pathologist’s ground truth segmentations. Several illustrative cases are shown in Fig. 2. However, we noticed that HEIP tends to make mistakes in detecting very large nuclei, by dividing them into smaller nuclei (Figure S1).

As instance segmentation is arguably the most influential step in the H&E image analysis, we evaluated the HEIP instance segmentation step with three metrics: segmentation quality (SQ), detection quality (DQ), and panoptic quality (PQ). See Methods section for more details. Two independent datasets were used to evaluate the segmentation performance of HEIP. The CellTypeValidation dataset was designed to assess instance segmentation performance across the cell types (neoplastic, inflammatory, connective, and epithelial). Results for different cell types are shown in Table 1. The best performance was observed in detecting neoplastic and epithelial cells, whereas the detection of connective and inflammatory cells was lower. Assessing all annotations without distinguishing between specific cell types, HEIP achieved a PQ of 0.75, DQ of 0.88, and SQ of 0.85 as shown in Table S3.

**Table 1:** Cell classification results in the CellTypeValidation dataset. The results of the cell classification of the 9461 train-in-the-loop annotated cells from the 20 ROIs extracted from H&E WSI of 19 HGSC samples are presented. The performance for the four cell types was evaluated using the Panoptic quality (PQ), detection quality (DQ), and segmentation quality (SQ) measurements. The confidence interval, denoted within parentheses, was calculated using a bootstrapped approach comprising 200 rounds. Bold font indicates higher values.

Cell type	PQ	DQ	SQ
Neoplastic cells	0.67 [0.624, 0.697]	0.77 [0.731, 0.811]	0.86 [0.849, 0.87]
Epithelial cells	**0.69 [0.615, 0.756]**	**0.79 [0.707, 0.874]**	**0.87 [0.846, 0.885]**
Connective cells	0.53 [0.454, 0.587]	0.64 [0.548, 0.71]	0.83 [0.818, 0.84]
Inflammatory cells	0.50 [0.424, 0.593]	0.59 [0.499, 0.69]	0.85 [0.844, 0.863]

The TumorSiteCellValidation dataset was designed to assess HEIP performance in different tumor sites (tubo-ovarian, omentum, and peritoneum), focusing on evaluating the performance across tumor–stroma interface. The results are presented in Table 2, which shows that HEIP has better performance in detecting neoplastic nuclei in omental tumors, achieving a PQ of 0.72, DQ of 0.80, and SQ of 0.90. Comparable results were observed in peritoneal tumors though performance was lower in the tubo-ovarian samples. Moreover, when evaluating overall annotations without distinguishing among various cell types, the instance segmentation results for the TumorSiteCellValidation dataset showed analogous outcomes to those of the CellTypeValidation dataset.

**Table 2:** Cell classification results in TumorSiteCellValidation dataset. The results of the cell classification for the dataset composed of 36 tumor–stroma interface ROIs of 18 H&E slides from different tissue types of HGSC patients are presented. Panoptic quality (PQ), detection quality (DQ), and segmentation quality (SQ) were considered for the evaluation. The confidence interval, denoted within parentheses, was calculated using a bootstrapped approach comprising 200 rounds.

	Tubo-Ovarian			Omentum			Peritoneum		
Cell type	PQ	DQ	SQ	PQ	DQ	SQ	PQ	DQ	SQ
Neoplastic cells	0.62 [0.487, 0.699]	0.70 [0.558, 0.791]	0.89 [0.87, 0.903]	0.72 [0.661, 0.783]	0.80 [0.736, 0.869]	0.90 [0.894, 0.905]	0.71 [0.654, 0.76]	0.80 [0.745, 0.859]	0.88 [0.875, 0.887]
Connective cells	0.54 [0.473, 0.608]	0.62 [0.544, 0.705]	0.86 [0.854, 0.876]	0.61 [0.556, 0.655]	0.70 [0.635, 0.752]	0.87 [0.865, 0.882]	0.66 [0.62, 0.7]	0.77 [0.725, 0.816]	0.86 [0.849, 0.871]
Inflammatory cells	0.45 [0.34, 0.547]	0.54 [0.404, 0.666]	0.76 [0.621, 0.845]	0.64 [0.595, 0.674]	0.75 [0.699, 0.794]	0.85 [0.842, 0.857]	0.56 [0.461, 0.646]	0.66 [0.549, 0.762]	0.84 [0.833, 0.857]

### Ploidy analysis

**Fig. 3:**
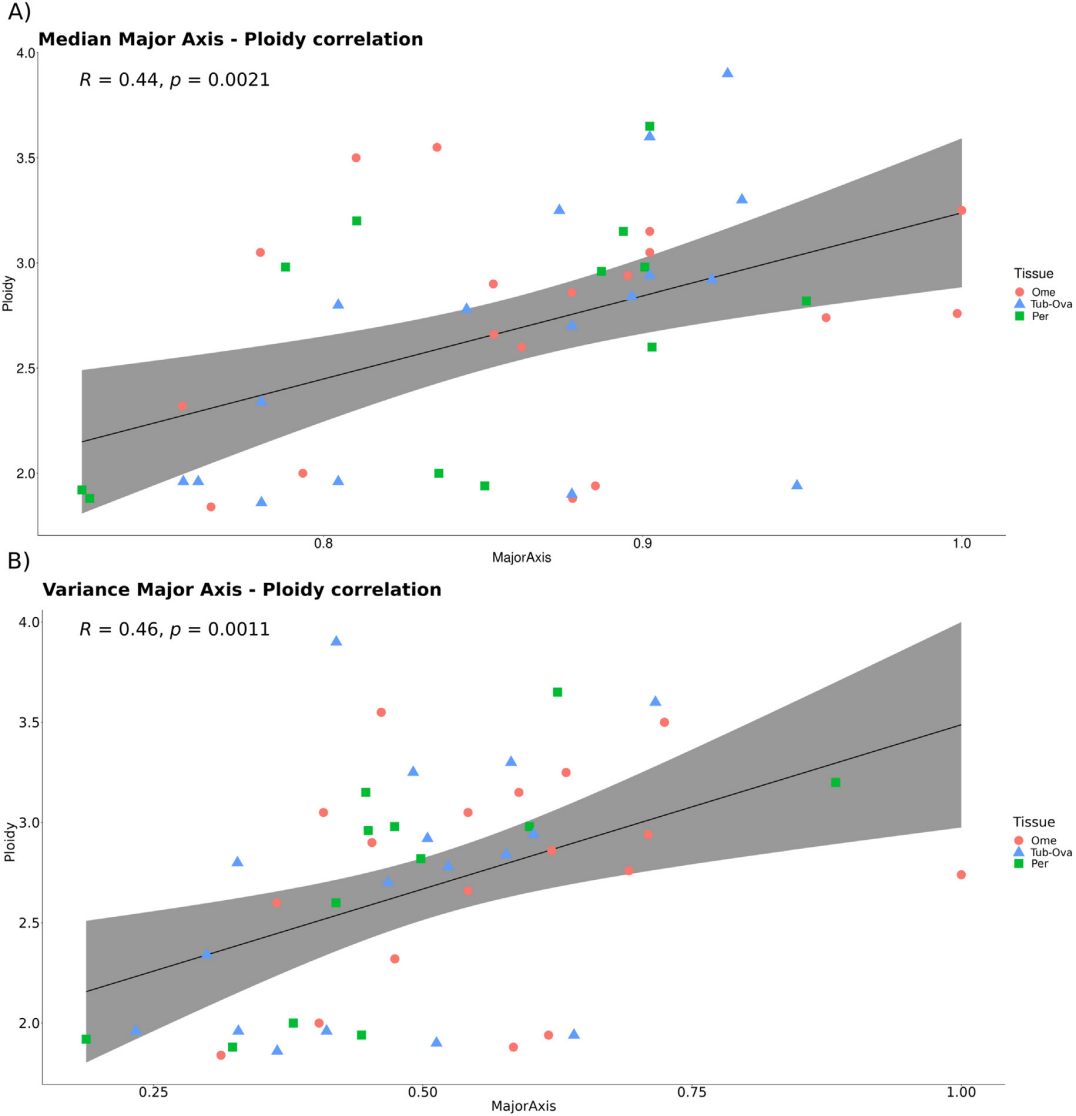
Correlation between major axis and ploidy values in ploidy association datates. The graph illustrates the correlation between ploidy values and major axis of nuclei across different tissues: omentum (18), peritoneum (12), and tubo-ovarian (17). The ploidy values and H&E image analysis were done using matched samples from the same section. Panel A: Each data point in the plot represents a sample for which we were able to correlate the median value of the major axis of nuclei with its respective ploidy value. Panel B: Each data point in the plot represents a sample for which we were able to correlate the variance value of the major axis of nuclei with its respective ploidy value. A clear positive correlation is observed for both panels. For clarity of understanding the tissue distribution, we have differentiated the three specific tissues in the graph: omentum (Ome), represented by red circles; tubo-ovarian (Tub-Ova), represented by light blue triangles; and peritoneum (Per), represented by green rectangles.

HEIP extracts several morphological features from cell nuclei, which can be utilized in downstream analyses. As an example, we explored the association between ploidy values computed from whole-genome sequencing data, as described in the Methods section, and the median and variance of four features of neoplastic nuclei extracted by HEIP (area, volume, major axis, and perimeter) in 47 samples. The sequencing data were generated from the same sample as the H&E images. The highest correlation was observed between the major axis and ploidy, in both median (0.44, *p* = 2.1 · 10^−3^) and variance (0.46, *p* = 1.1 · 10^−3^), as shown in Table 3. Overall, ploidy and major axis, are associated among samples from the same patient (Fig. 3).

**Table 3:** Correlation results between morphological features and ploidy, in ploidy association dataset. The table shows the correlation values between the morphological features extracted from the H&E samples and the genomic ploidy value of the exact section, in particular the median and the variance of area, volume, major axis, and perimeter, with the respective ploidy value of the samples. The first two columns display the correlation results of all 47 samples, whereas the latest two columns exclude the two samples with unconventional tissue structure. Bold font indicates higher values.

	Correlation with ploidy		Correlation with ploidy (after outliers deletion)	
Features	Median	Variance	Median	Variance
Area	0.24 (*p* = 0.1)	0.29 (*p* = 4.7 · 10^-2^)	0.36 (*p* = 0.02)	0.33 (*p* = 2.6 · 10^−2^)
Volume	0.21 (*p* = 0.16)	0.26 (*p* = 7.8 · 10^-2^)	0.32 (*p* = 0.03)	0.30 (*p* = 4.7 · 10^−2^)
Major axis	**0.44** **(*p* = 2.1 · 10**^**−3**^**)**	**0.46** **(*p* = 1.1 · 10**^**-3**^**)**	**0.5** **(*p* = 5 · 10**^**−4**^**)**	**0.42** **(*p* = 4.4 · 10**^**−3**^**)**
Perimeter	0.34 (*p* = 0.02)	0.41 (*p* = 4.5 · 10^-3^)	0.44 (*p* = 2.2 · 10^−3^)	0.38 (*p* = 1.1 · 10^−2^)

The correlation between ploidy and major axis was moderate, and there are some outliers that we inspected in a more detailed fashion. Two samples (EOC465_pPer1 and EOC557_pOme1) exhibit technical tissue artifacts, featuring stretched tissues, and elongated cells that have been incorrectly segmented and classified (Fig. S2). Eliminating these 2 outliers, the correlation increased for the median to 0.5 (*p* = 5 · 10^−4^), while for the variance, it decreased slightly to 0.42; however, the correlation remained statistically significant (*p* = 4.4 · 10^−3^). Genomic ploidy values and anatomical tumor locations do not correlate (ANOVA; *p* = 0.93, Fig. S3).

## Discussion

Digitalized H&E slides are becoming increasingly important in cancer research.^1^^,^^34^ Herein, we have presented HEIP, an automated pipeline for processing H&E images, detecting cell types, and extracting morphological features of the cells, as well as cell percentages and Shannon Index. HEIP is designed and implemented as modular software and trained for HGSC H&E images. Modularity ensures versatility of HEIP to various image analysis tasks with minimal modifications required. Furthermore, the modular design permits easy upgrading to more sophisticated methods as they become available. The output of the nuclei detection is a json file, which contains the polygons with the coordinates of each detected nucleus. By using json files, HEIP reduces the need for memory, compared to the image masks, and storage space, making it efficient.

We showed the utility of HEIP in the analysis of H&E images from histopathological research samples of HGSC patients. Importantly, HEIP estimations for cell type annotations (neoplastic, inflammatory, connective, and epithelial nuclei) agreed well with the pathologist’s ground-truth annotations. However, HEIP did not accurately recognize the borders of very large nuclei and tended to divide them into several nuclei. In general, HEIP performance is higher (neoplastic) or on par (connective and inflammatory) with the other nuclei segmentation methods trained with the PanNuke dataset,^21^ whose PQ values range from 0.3 to 0.5. The recognition of dead cells was not involved in the analyses as the number of dead cells in the datasets was negligible.

As an example of a downstream analysis, we explored correlation between ploidy and nuclear morphological features using WGS and histomorphological data from the same tumor piece. Our results indicate a significant moderate correlation between major axis of neoplastic nuclei and ploidy. These findings are consistent with Boehm et al.,^7^ who reported a possible association between nuclear size and WGS.

The utilization of an automated pipeline, such as the HEIP, for cell nuclei recognition and feature extraction can offer significant improvements in both accuracy and efficiency of histological image analysis. This, in turn, can lead to numerous advantages in clinical routine and open new avenues for research. By eliminating the need for manual annotation of image features, the variability and bias of the analysis can be reduced, as well as enhancing reproducibility. Furthermore, the use of automated pipelines allows for the rapid analysis of whole-slide images, enabling easier evaluations of the sample's morphology and cell composition. This approach has the potential to enhance our understanding of complex biological systems, ultimately improving the diagnosis and treatment of cancer.

Taken together, we have developed an open-source pipeline HEIP for comprehensive analysis of H&E images. We have shown the utility of HEIP in detecting selected cell types and nuclear morphological features in HGSC H&E images. As HEIP is modular, it can be modified to accommodate H&E images from other cancers as well.

## Limitations of the study

The primary limitations of HEIP regards the instance segmentation phase. HEIP faces challenges in accurately segmenting very large nuclei, often resulting in their over-segmentation into smaller entities. Furthermore, although HEIP demonstrates promising performance in classifying neoplastic cells, it encounters difficulties in accurately identifying dead cells as neoplastic. Future work would incorporate more dead cell annotations in the training set.

## Declaration of Competing Interest

The authors declare that they have no known competing financial interests or personal relationships that could have appeared to influence the work reported in this paper.

## Data Availability

All raw DNA sequencing data is submitted to the European Genome-phenome Archive (EGA) and will be publicly available under study accession number EGAS00001006775. The code and the documentation of HEIP are available at https://github.com/ValeAri/HEIP.

## Appendix A Supplementary data

Supplementary material Image 1
