# Supplementary material for: H&E image analysis pipeline for quantifying morphological features
Source: J Pathol Inform. 2023 Oct 5;14:100339. doi: 10.1016/j.jpi.2023.100339 (PMC10616375; doi:10.1016/j.jpi.2023.100339)
Supplement: Supplementary file 1 — Supplementary material [file mmc1.docx]

# **Supplementary Tables and Figures**

| Post-Process Method | PQ |
| --- | --- |
| Omnipose | 0.75 [0.72, 0.769] |
| HoVer-Net | 0.73 [0.667, 0.764] |

**Table S1. Post-processing method results.** The binary PQ values for HoVer-Net and Omnipose post-processing across 20 ROIs (1000x1000px and a total of 9,461 annotations) validation images extracted from H&E WSI of 19 HGSC samples. The confidence interval, denoted within parentheses, was calculated using a bootstrapped approach comprising 200 rounds.

| Area | Number of pixels occupied by a cell nucleus |
| --- | --- |
| Volume | Approximate volume of a cell nucleus, modeled as an ellipsoid or sphere |
| Solidity | Convexity or compactness of a cell nucleus |
| Eccentricity | Degree of elongation of a cell nucleus compared to a perfect circle |
| Minor Axis | Width of a cell nucleus |
| Major Axis | Length of a cell nucleus |
| Aspect Ratio | Ratio of the height to the width of a cell nucleus |
| Perimeter | Length of the boundary of a cell nucleus |
| Cell Percentage | Percentage of each cell type within a tissue |
| Shannon Index | Measure of the diversity of species (here, neoplastic, inflammatory and connective cell types) |

**Table S2. Extracted nuclei features.** List of the extracted features for the cell nuclei with their definition.

| Validation Dataset | PQ | DQ | SQ |
| --- | --- | --- | --- |
| CellTypeValidation | 0.75 [0.721, 0.768] | 0.88 [0.858, 0.898] | 0.85 [0.833, 0.857] |
| TumorSiteCellValidation | 0.77 [0.756, 0.782] | 0.88 [0.869, 0.894] | 0.87 [0.866, 0.878] |

**Table S3. Instance segmentation results for both CellTypeValidation and TumorSiteCellValidation datasets.** The instance segmentation outcomes for both the CellTypeValidation and TumorSiteCellValidation datasets, without differentiation based on cell types or tissues, were presented. The assessment of overall annotations is conducted using the Panoptic Quality (PQ), Detection Quality (DQ), and Segmentation Quality (SQ) metrics. The confidence interval, denoted within parentheses, was calculated using a bootstrapped approach comprising 200 rounds.

**Figure S1. Segmentation mistake of a very large cell nucleus.** **Panel A.** ROI highlighted with a black circle, showing the large size of the nucleus. **Panel B.** Incorrect HEIP segmentation, only a small portion of the nucleus is recognized as cell material. **Panel C.** Correct border of the large cell nucleus.
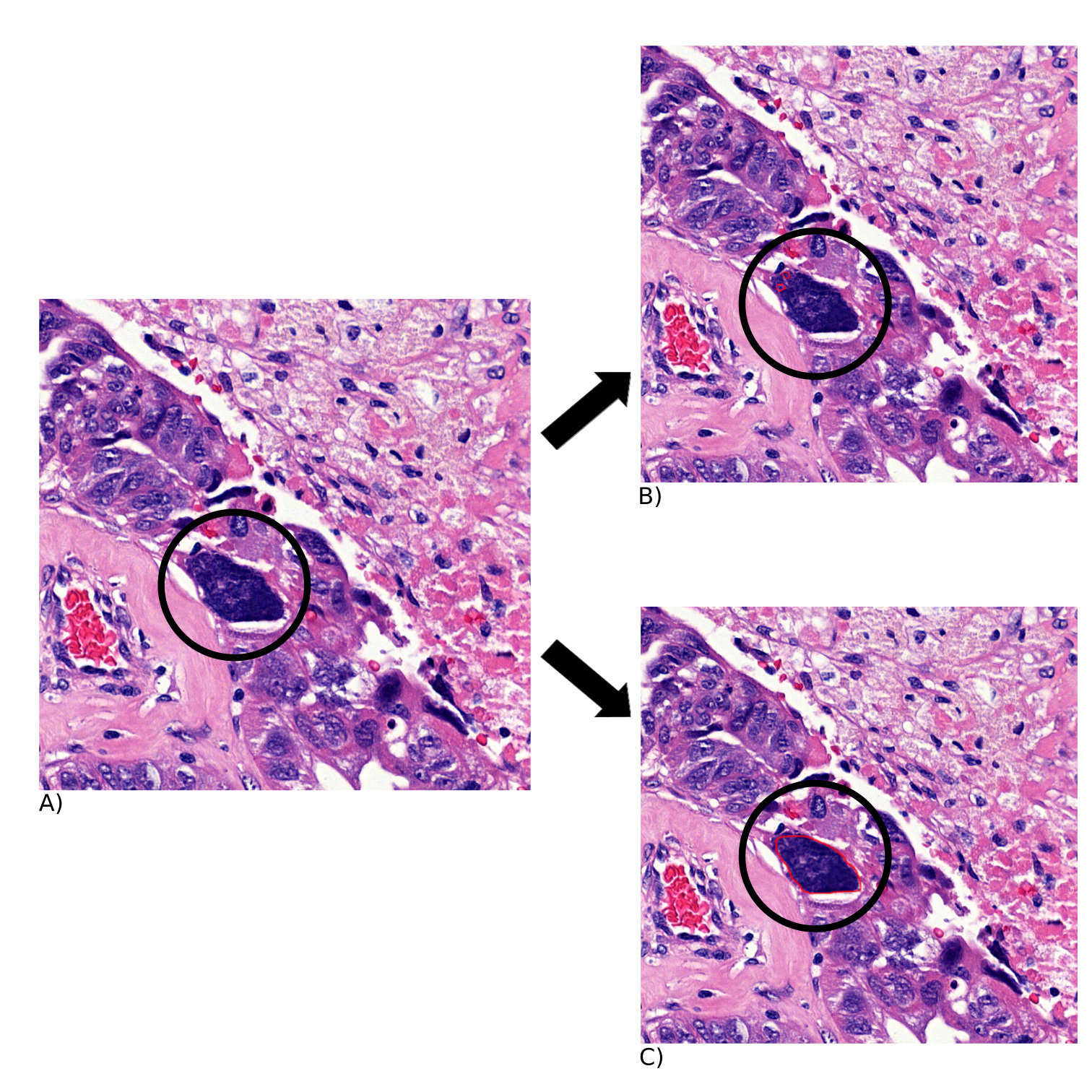


**Figure S2. Tissue with technical artifact in EOC465_pPer1 sample.** **Panel A.** a portion of the WSI from peritoneum, with elongated cell nuclei and stretched tissue. **Panel B.** Zoomed version of the tissue.
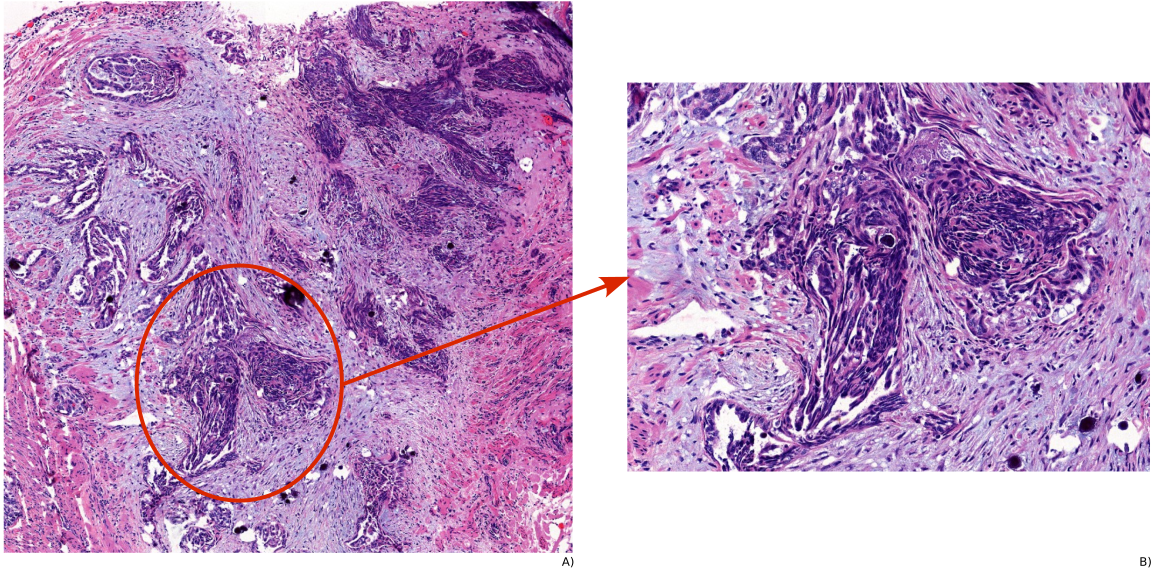


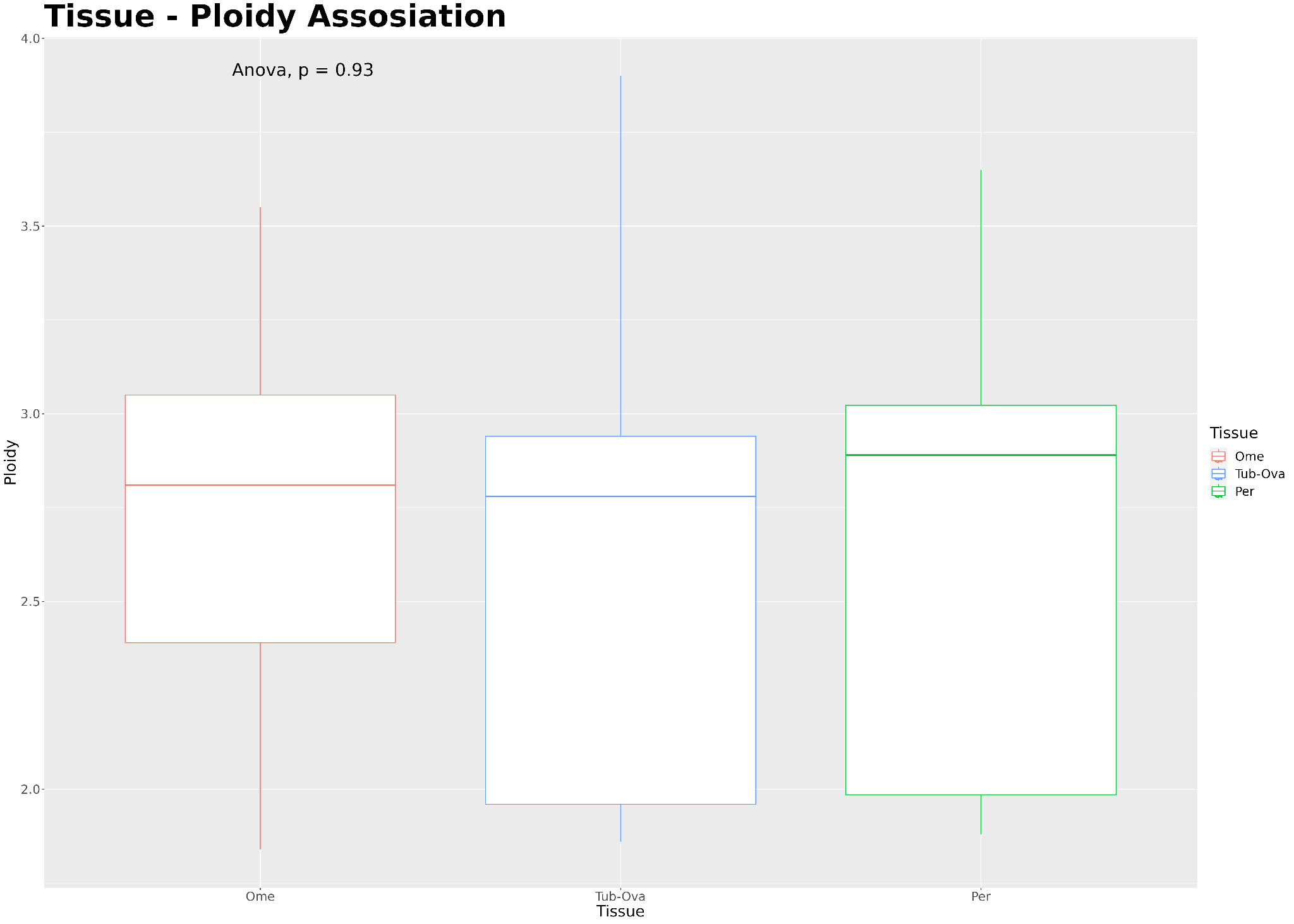
**Figure S3. Association between tissue sections and ploidy value.** This figure illustrates the relationship between various tissue sections, including omentum (Ome) in red, tubo-ovarian (Tub-Ova) in light blue, and peritoneum (Per) in green. The corresponding ploidy values for each tissue section are displayed using boxplot representations. Additionally, the ANOVA analysis results are presented atop the graph, indicating that no significant correlation is observed among the variables.
